# Fatalism in the Early Days of the COVID-19 Pandemic: Implications for Mitigation and Mental Health

**DOI:** 10.3389/fpsyg.2021.560092

**Published:** 2021-06-28

**Authors:** Joseph Hayes, Laura Clerk

**Affiliations:** Department of Psychology, Acadia University, Wolfville, NS, Canada

**Keywords:** COVID-19, fatalism, social distancing, mental health, media messaging

## Abstract

This research assessed fatalism toward COVID-19 and its role in behavioral intentions to support mitigation efforts (e. g., social distancing) and mental well-being. A COVID-19 fatalism measure was developed, and a messaging manipulation (fatalistic vs. optimistic vs. no message) was created to examine causal links between fatalism scores. Support for mitigation efforts and negative affect (anxiety, fear, depression, and insecurity) were measured to examine the consequences of fatalism toward COVID-19. Results showed that the fatalistic messaging condition increased fatalism whereas the optimistic message reduced it. The effects of the messaging manipulation were also apparent in the downstream measures of support for mitigation and negative affect through the mediator of fatalism toward COVID-19. Specifically, fatalism negatively predicted intentions to support mitigation. Regarding mental health, fatalism was positively associated with depression but negatively associated with fear and insecurity. Implications for COVID-19 mitigation efforts and mental health in the face of the coronavirus pandemic are discussed.

## Fatalism in the Fight Against COVID-19: Implications for Mitigation and Mental Health

On March 11th, 2020, the World Health Organization declared the COVID-19 outbreak a global pandemic (World Health Organization, 2020). In the 12 months that followed, more than 100 million people were infected and nearly 3 million people died (Worldometer, 2020). In the early days of this pandemic, most governments attempted to stop the spread of the virus by instituting lock-down measures and other mitigation protocols. Schools and businesses were closed, and people were asked to stay home as much as possible, practice social distancing when in public, and avoid large gathering or crowded environments (Centers for disease control prevention, 2020). Despite these efforts, the global spread of COVID-19 could not be contained.

As the death-toll from the pandemic rose, so too did the mental toll associated with mitigation efforts. Many people lost their job and faced severe financial strain (Mutikani, 2020; United States Department of Labor., 2020). Many more shifted to working from home, often in cramped urban apartment spaces, while also struggling to manage childcare and homeschooling (Cooney, 2020). Incidents of domestic violence increased (Taub, 2020). And more generally, people were forced to forego many of the things that typically provide meaning and purpose to life (e.g., social contacts, freedom of movement, sports and entertainment). To make matters worse, it quickly became apparent that this would likely be the new normal until a vaccine could be developed, which was estimated to take a year or more (Boyle, 2020).

Although the hardships and devastation of the pandemic would increase substantially as it wore on, the initial shock and disruption already seemed nearly impossible to manage during the early days. Rather than continue to struggle with mitigation efforts, we reasoned that many people would be willing to give up and submit to the deadly pandemic rather than fight against it. Given that most cases of COVID-19 are relatively mild, the appeal of letting go, and allowing the virus to wash through the population may have seemed more attractive than maintaining efforts to mitigate its spread. Plus, given the invisible nature of the virus (i.e., you cannot directly observe it in the environment), uncertainty regarding how it is transmitted (Han et al., 2020), and the delayed effects of mitigation efforts, it can be easy to become fatalistic and feel that nothing can be done to stop the virus from spreading. The purpose of the current research was to assess fatalism toward COVID-19 at a pivotal point in America's efforts to fight the virus—the first few weeks into the pandemic. Moreover, we aimed to examine the effect of different media messages on feelings of fatalism, and sought to gauge the consequences of COVID-19 fatalism on support for continued mitigation efforts and mental well-being.

### Fatalism Toward COVID-19

Fatalism is the belief that one's actions have little or no significant impact on important outcomes (Zimbardo and Boyd, 1999). People who are high in fatalism tend not to engage future-oriented planning, expend little effort in trying to achieve desirable goals, and are generally resigned to fate. In other words, they are willing to let external forces take over. Although the tendency to display fatalism is an individual difference, there are also situational circumstances that will promote fatalistic thinking independently of differences in personality. Indeed, fatalism may be particularly likely during the COVID-19 pandemic. Virus particles are invisible and easily transmitted from person to person. Those who contract the virus often have no idea where they became infected. Indeed, in some cases, transmission of the virus can happen in the absence of symptoms. The pandemic represents a powerful external force that can easily lead people to conclude that there is nothing they can do to influence the situation. In other words, they may feel that how the pandemic turns out is largely up to fate.

### Mental Health

When fatalism is pervasive, it tends to be strongly associated with depression and hopelessness (Seligman, 1975; Zimbardo and Boyd, 1999). However, fatalistic beliefs can also function to reduce the fear and anxiety aroused by insurmountable threats (Hayes et al., 2016; Lifshin et al., 2020). Struggling to control outcomes that appear intractable triggers anxious motivational conflict (Carver and Scheier, 1998; Gray and McNaughton, 2000), while choosing to let go of them can reduce this anxiety by eliminating the tension produced by wanting to control something uncontrollable (Rothbaum et al., 1982; Hayes et al., 2017).

With respect to COVID-19, mitigation efforts are difficult, costly, and have no clear end-date. Thus, becoming fatalistic in the fight against COVID-19 can be an attractive way of reducing concerns about the pandemic. Indeed, recent work by Lifshin and colleagues suggests that people can become motivated to feel helpless against the virus to justify inaction and reduce anxiety (Lifshin et al., 2020). Importantly, however, we maintain that this method of palliation can also have broader negative consequences for mental health. When fatalism is extensive, it can promote depression and generalized disengagement from life (Hayes et al., 2016, 2017).

### Commitment to Mitigation Efforts

Another trouble with fatalism is that it reduces motivation and planful self-regulation (Hayes et al., 2016), which may be especially problematic vis-à-vis COVID-19 because it may undermine the principal means of addressing the pandemic—namely, social distancing. Becoming fatalistic about COVID-19 may lead people to ignore public health recommendations (e.g., “*we're all going to get this virus anyway, so why stay at home and suffer?*”), which is dangerous for the general public as well as the fatalistic individual as it increases the risk that they will become infected and spread the virus by not taking proper precautions. Understanding factors that contribute to COVID-19 fatalism, and how we can reduce these factors, is therefore imperative if collective mitigation efforts are to be successful.

### Media Messaging

One factor that may be particularly important in creating fatalism toward COVID-19 is the way in which it is presented in the media. Indeed, nearly every news story now appears related to the pandemic in some manner or another, and the information is often dire or shocking. While many of these messages seek to affirm the importance of collective action and the ironic sense of community that can come from social distancing for the well-being of others (e.g., #AloneTogether; Harrop, 2020), other messages often directly promote fatalism by claiming that the spread of the disease is inevitable (Slaughter, 2020). These messages often voice concern about the long-term economic impact of staying at home and shuttering businesses, suggesting that the cost associated with continued mitigation efforts is far greater than the cost of the virus (Hilton, 2020; Singer and Plant, 2020). U.S. President Donald Trump appeared to share these concerns when tweeting on March 23rd “WE CANNOT LET THE CURE BE WORSE THAN THE PROBLEM ITSELF” (Trump, 2020). Although these messages are very clearly anti-mitigation, we suspect they may be most effective in reducing support for mitigation efforts when they instill a sense of fatalism toward COVID-19. Indeed, fatalism and inaction go hand-in-hand.

### Study Overview

The purpose of the current research was to assess levels of fatalism toward COVID-19, to understand what factors influence this construct, and to examine the consequences of fatalism for mental health and support for COVID-19 mitigation efforts. Accordingly, we developed a self-report measure of fatalism toward COVID-19 and collected an online survey. To assess how COVID-19 fatalism can be causally influenced, we designed a fatalistic message arguing that the pandemic is unstoppable and that mitigation efforts may do more harm than good (cf., Hilton, 2020; Singer and Plant, 2020; Slaughter, 2020; Trump, 2020). The fatalistic message intended to mimic those presented in the media or shared by prominent figures that the general public has been exposed to throughout the pandemic. For comparison, we created an optimistic message that emphasized the effectiveness of mitigation efforts and the connectedness that can come from tackling the pandemic collectively. We expected the fatalistic message to increase fatalism and the optimistic message to reduce it.

To assess the consequences of COVID-19 fatalism, at the end of the study we assessed support for mitigation efforts and negative emotionality. Regarding support for mitigation, we expected fatalism to be associated with reduced support for these efforts. Moreover, we hypothesized that our fatalistic and optimistic messaging conditions would influence support for mitigation by virtue of affecting self-reported feelings of fatalism (i.e., mediation). Regarding negative emotion, we expected fatalism to be positively associated with depression (Zimbardo and Boyd, 1999), but negatively associated with anxiety given evidence that fatalism in the face of insurmountable threats can reduce anxiety (Hayes et al., 2016; Lifshin et al., 2020).

## Method

### Participants and Design

In keeping with open science practices, we report all measures and manipulations included in the study. Full study materials are available online at https://osf.io/sx7g2/. We also explain how sample size was determined and report all data exclusions.

To determine minimum sample size requirements to confidently test our hypotheses, we conducted an a priori power analysis using G^*^Power (Faul et al., 2007), and sought at least 80% power (with an alpha of 0.05) to detect a small effect (i.e., *f* = 0.10–0.24; *d* = 0.20–0.49). The number of participants required to detect the lowest end of this range (*f* = 0.10; *d* = 0.20) using these criteria in a one-way ANOVA with three conditions yielded the highest estimate (*N* = 969), so we strove to obtain a sample size that approximated this number.

Participants were 1,025 people recruited online through Amazon's Mechanical Turk. They were randomly assigned to one of three conditions in a between-subjects design. The only requirement for participation was United States residence. Exclusions included 149 participants who failed an attention check item asking them to leave a question blank (i.e., *Please do not answer this question, it is here to see if you are paying attention*.), 19 who did not correctly answer at least two (of four) multiple-choice questions about the contents of the article that they read, and six others for failing to complete all dependent variables for our main analyses. The total number of participants after exclusions was 851 (fatalistic *n* = 274, optimistic *n* = 291, no message *n* = 286), which fell short of our sample size goal. Nevertheless, a *post-hoc* sensitivity analysis revealed that we retained 80% power to detect small effects (*f* = 0.11, *d* = 0.21; and 95% power to detect *f* = 0.13, *d* = 0.27), so we were confident in proceeding with our analyses without collecting more data. The final sample ranged in age from 18 to 78 (*M*_age_ = 41.0, *SD*_age_ = 14.0), and gender balance was roughly equal (female = 443, male = 391, other = 5, prefer not to disclose = 6).

### Measures and Procedure

The study was conducted on March 27th, 2020, 11 days into the initial mitigation period aimed at providing 15-days to slow the spread of the novel coronavirus (WhiteHouse.gov, 2020). The study was reviewed by an institutional research ethics board and was deemed to pose no more than minimal risk. Participants were informed that the study was an investigation of personality, attitudes, and opinions. They were not told that the purpose of the study was related to COVID-19 until the debriefing. Upon consenting to participate, respondents began by complete a series of demographic questions, followed by three brief personality questionnaires.

### Demographic and Personality Variables

Demographic items included age, gender, household income, education, and political orientation (among others, see online supplement for complete list of demographic items). We measured three personality factors that seemed like plausible candidates for influencing fatalism. Specifically, we assessed self-esteem (Rosenberg, 1965) and trait sensitivity to rewards and punishments (BAS and BIS; Carver and White, 1994) given that these variables influence reactions to threat (see Pyszczynski et al., 2004; Jonas et al., 2014). Exploratory analyses controlling for demographic and personality factors and exploratory tests of moderation are presented in the Supplementary Online Material (SOM; see https://osf.io/sx7g2/).

### Messaging Condition

Participants were randomly assigned to one of three messaging conditions. In the control condition, participants read no message and simply proceeded to a series of questions related to their attitudes and opinions about the COVID-19 pandemic. By contrast, those in the fatalistic and optimistic messaging conditions read a brief opinion piece before proceeding to these questions. The essays began with a threatening paragraph outlining the severity of the COVID-19 pandemic:

*COVID-19 is a killer. It has already killed more than 25,000 people worldwide and will likely kill many hundreds of thousands more. Making things worse, it is an invisible killer. We cannot see it, and we cannot even know when we have it on our hands. The only way that we know to keep it at bay is to stay away from each other. Social (or physical) distancing measures have been in place across the Western World for nearly 2 weeks. But the spread of COVID-19 rages on*.

For participants in the *fatalistic condition*, the essay went on to describe how social distancing can only be a temporary fix, and that the virus will remain problematic until we develop a vaccine, which will not happen for 18–24 months. The article then struck a fatalistic chord by asking whether people are truly willing to engage social distancing for 2 years. The author indicates that the virus is unstoppable and that he would rather let it run its course so that we can get back to normal sooner than later.

Participants in the *optimistic condition* read an article that began with the same opening paragraph but then proceeded to argue that social distancing is effective. The author pointed toward China and South Korea as examples of its effectiveness. The message is optimistic but nevertheless realistic, suggesting that social distancing will not eradicate the virus but will buy time so that a vaccine can be developed within 18–24 months. The author concludes by indicating that he is willing to do his part to prevent the spread of the virus. Finally, he appeals to the togetherness that collective social distancing can offer (see SOM for the full text of both messaging conditions).

Participants in the fatalistic and optimistic conditions then proceeded to complete five simple reading comprehension questions that were included to ensure adequate processing of the message. Four of these questions were multiple-choice, whereas one was an open-ended item asking participants to indicate the overall theme of the article. Only participants who correctly answered at least two of the four multiple choice questions were retained for data analyses.

### Specific Worries

Next, participants completed a 7-item scale assessing their specific worries related to the COVID-19 crisis. These items were included for exploratory purposes. They assessed worries about death and finances for the self, close others, and strangers. The final item assessed concern for the economy (see SOM for exploratory analyses of these items).

### Fatalism Toward COVID-19

Participants then completed a 16-item scale assessing fatalism toward COVID-19 (α = 0.94; see Table 1 for complete item-details; see SOM for factor analytic results and other exploratory analyses). This was our main dependent variable, and consisted of seven positively keyed items (“*Since whatever will be will be, it doesn't really matter what I do to try to stop COVID-19*”) and nine negatively keyed items (indicative of self-efficacy; e.g., “*It is within my power to help reduce the spread of COVID-19*”). Participants rated their agreement with each item using a 7-point Likert scale (1 = strongly disagree; 7 = strongly agree).

**Table 1:** Descriptive statistics for scale items assessing fatalism toward fighting Covid-19.

**Item**	**Mean**	**SD**	**Skewness**	**Item-total Correlation**
Staying home can make all the difference in the fight against covid-19*.	5.70	1.28	−1.35	−0.74
I can help to stop the spread of covid-19*.	5.52	1.32	−1.24	−0.73
I believe that helping to stop covid-19 is within my control*.	5.37	1.38	−0.97	−0.73
My actions can contribute to stopping the spread of covid-19*.	5.61	1.33	−1.27	−0.73
Since whatever will be will be, it doesn't really matter what I do to try to stop covid-19.	2.42	1.68	1.14	0.72
I have the ability to make decisions that will reduce the spread of covid-19*.	5.68	1.29	−1.39	−0.72
What I do now to fight covid-19 matters in the long run*.	5.64	1.36	−1.25	−0.71
I often feel that there is no point in even trying to stop the spread of covid-19.	2.43	1.69	1.09	0.70
It is within my power to help reduce the spread of covid-19*.	5.46	1.37	−1.13	−0.69
When thinking about tackling covid-19, I often think “why bother?”	2.35	1.66	1.24	0.68
My actions will make a difference in reducing the death-toll from covid-19*.	5.41	1.38	−1.13	−0.68
It doesn't make sense to worry about covid-19 because there is nothing that I can do about it anyway.	2.74	1.72	0.88	0.67
Social distancing is NOT a good way to fight covid-19.	2.17	1.52	1.49	0.66
There is no effective way to stop covid-19 from spreading.	3.03	1.68	0.75	0.60
Forcing people who are not sick into self-isolation will reduce the spread of covid-19*.	5.49	1.50	−1.11	−0.58
The spread of covid-19 is controlled by forces that I cannot influence.	4.06	1.73	−0.12	0.45

SD, Standard Deviation; Items followed with an asterisk (*) are reverse-keyed.

### Behavioral Intention to Support Mitigation Efforts

Immediately after the fatalism questionnaire, we included an 11-item scale assessing behavioral intentions to support mitigation efforts (α = 0.86, see SOM for complete item-details). Participants again responded using the 7-point scale. The items were geared predominantly toward intentions to engage social distancing (e.g., *I plan to keep my distance from others*) and to remain isolated (e.g., *I plan to stay isolated for as long as it is required*), but also assessed support for mitigation efforts more broadly (e.g., *I support lockdown efforts aimed at reducing the spread of COVID-19*).

### Emotional Distress

Finally, after rating their support for mitigation efforts, participants completed a brief emotion measure to gauge their emotional well-being. The measure consisted of 20 items assessing four different emotions (5-items each): anxiety (α = 0.84), fear (α = 0.95), depression (α = 0.93), and insecurity (α = 0.87; see SOM for complete item-details). Although our hypotheses were specifically related to anxiety and depression, we included items assessing fear and insecurity for exploratory purposes. Participants rated the extent to which they were currently experiencing these emotions using a 7-point scale (1 = not at all; 7 = very much). Upon completion, participants were thanked for their participation and fully debriefed.

## Results

Deidentified data and analysis script for all analyses reported below are available online at https://osf.io/sx7g2/. A document containing supplemental exploratory analyses that accompany the main findings can be found by following the same link.

Prior to beginning our analyses, we reversed scored negatively keyed items and computed scale means for each of our dependent variables. Descriptive statistics for the global sample are displayed in Table 2.

**Table 2:** Descriptive statistics for dependent variables.

	**Descriptive statistic**			
**Dependent variable**	**Mean**	**SD**	**Skewness**	**Reliability**
COVID-19 Fatalism	2.58	1.07	0.76	0.94
Support for Mitigation Efforts	5.51	1.00	−0.79	0.86
Anxiety	2.98	1.44	0.46	0.84
Fear	2.62	1.67	0.90	0.95
Depression	2.69	1.66	0.84	0.93
Insecurity	3.77	1.44	0.01	0.87

*SD, Standard Deviation; Reliability, Cronbach's α*.

### Fatalism Toward COVID-19

**Figure 1:**
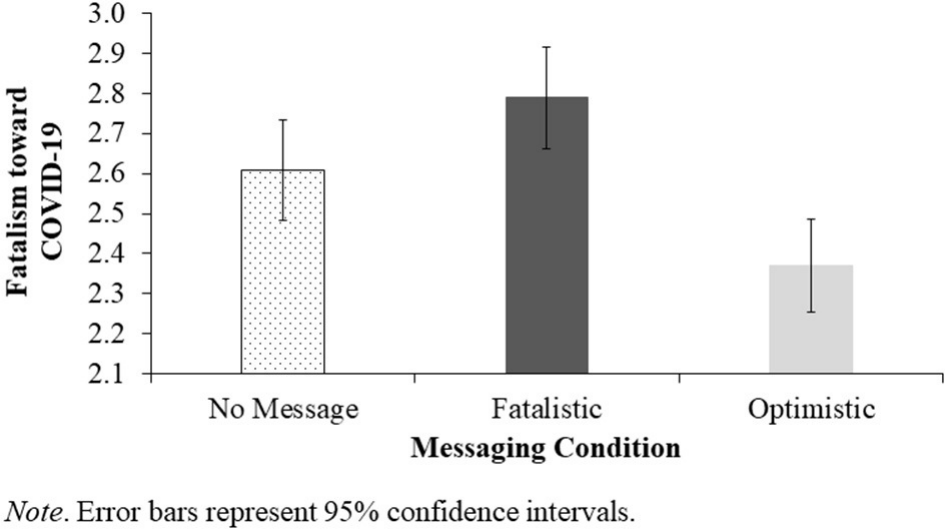
Effect of Messaging Condition on Fatalism toward COVID-19.

To test our hypothesis about the effect of messaging condition on fatalism toward COVID-19, we conducted a one-way between-subjects ANOVA on the fatalism scores. Results showed a significant effect of condition, *F*_(2, 848)_ = 11.16, *p* < 0.001, η^2^ = 0.03 (see Figure 1). Consistent with hypotheses, pairwise comparisons revealed that the fatalistic message increased fatalism relative to the no message control condition, *t*(848) = 2.04, *p* = 0.041, *d* = 0.17, whereas the optimistic message reduced fatalism relative to no message, *t*(848) = −2.68, *p* = 0.007, *d* = −0.22.

### Behavioral Intentions to Support Mitigation Efforts

Our next analysis examined the consequences of COVID-19 fatalism for behavioral intentions to support mitigation efforts. First, a bivariate correlation between these variables showed a highly significant association, *r*(851) = −0.78, *p* < 0.001. Thus, higher levels of fatalism toward COVID-19 were associated with lower behavioral intentions to support mitigation efforts.

**Figure 2:**
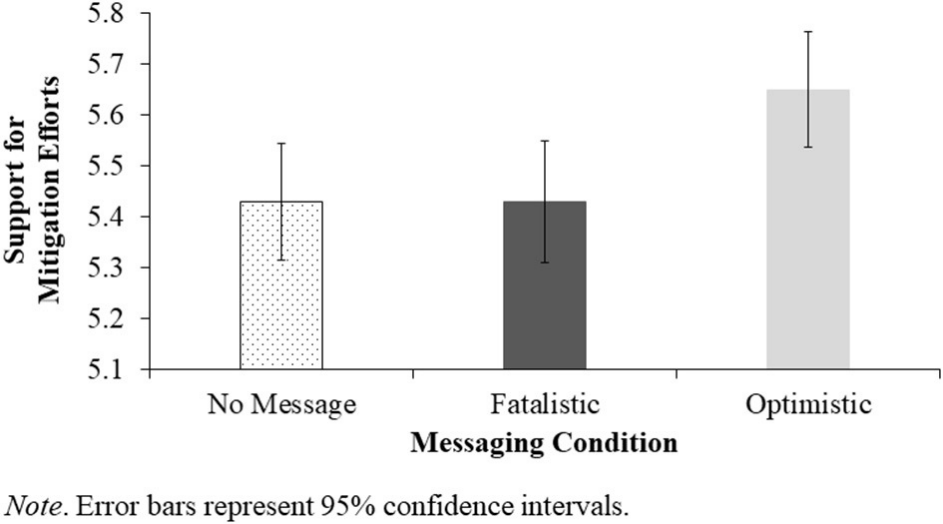
Effect of Messaging Condition on Support for COVID-19 Mitigation Efforts.

We also examined the effect of our messaging manipulation on support for mitigation efforts. A one-way ANOVA on support for mitigation with message condition as the independent variable revealed a significant effect, *F*_(2, 848)_ = 4.33, *p* = 0.013, η^2^ = 0.01 (see Figure 2). Pairwise comparisons revealed that whereas the optimistic message increased support for mitigation efforts relative to no message, *t*(848) = 2.57, *p* = 0.010, *d* = 0.21, the fatalistic message had no effect on support for mitigation, *t*(848) = −0.00, *p* = 0.997, *d* = −0.00.

**Figure 3:**
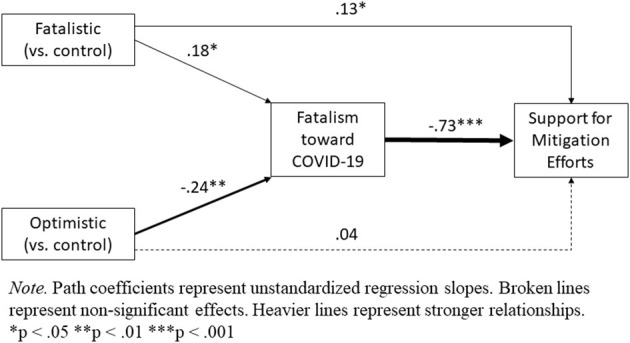
Path Model of Indirect and Direct Effects of Messaging Condition on Support for Mitigation Efforts Through the Mediator of Fatalism toward COVID-19.

Our main hypothesis regarding the effect of messaging condition on support for mitigation was that it would be mediated by fatalism toward COVID-19. Thus, we tested the indirect effect of our messaging manipulation on support for mitigation efforts through the hypothesized mediator of fatalism. Accordingly, we used Hayes' (2018) PROCESS macro to regress support for mitigation on messaging condition (dummy-coded to compare the fatalistic message with control in code 1 and the optimistic message with control in code 2) through the mediator of COVID-19 fatalism (Model 4, 5,000 bootstrap resamples). This analysis showed that the fatalistic message reduced support for mitigation indirectly by increasing fatalism toward COVID-19, *b* = −0.13, 95% confidence interval (CI) [−0.266, 0.003], whereas the optimistic message increased support for mitigation by reducing fatalism, *b* = 0.17, 95% CI [0.051, 0.299]. After accounting for these indirect effects, messaging condition still exerted a significant direct effect on support for mitigation efforts, *F*_(2, 847)_ = 3.31, *p* = 0.037, $\eta_{p}^{2}$ = 0.003. Interestingly, pairwise comparisons showed that whereas the direct effect of the optimistic message was not significant, *t*(847) = 0.77, *p* = 0.443, *d* = 0.04, the fatalistic message now revealed a significant direct effect, *t*(847) = 0.2.52, *p* = 0.012, *d* = 0.13, such that participants *increased* their support for mitigation efforts after reading the fatalistic message relative to no message (see Figure 3 for a full path model).

### Emotional Distress

Finally, we examined the emotional consequences of fatalism toward COVID-19. Bivariate correlations between fatalism and each of the negative emotions measured in the study are presented in Table 3. Given research showing divergent unique associations between fatalism and anxiety and depression when these highly correlated negative emotions are covaried for each other in statistical analyses (see Hayes et al., 2016; Hayes and Hubley, 2017), we also examined partial correlations between fatalism and each negative emotion controlling for the others in Table 3. In summary, although the overall correlations show only a significant positive association between fatalism and depression, the partial correlations also show significant *negative* associations between fatalism and fear and insecurity.

**Table 3:** Bivariate correlations among fatalism and negative emotions.

**Variable**	**1**	**2**	**3**	**4**
Anxiety (1)	–			
Fear (2)	0.80^***^	–		
Depression (3)	0.79^***^	0.81^***^	–	
Insecurity (4)	0.26^***^	0.27^***^	0.33^***^	–
Fatalism	0.07	0.02	0.09^**^	−0.05
Fatalism (partial correlations)	0.04	−0.10^**^	0.12^***^	−0.08^*^

*Partial correlations with fatalism (bottom line) represent relationships between fatalism and each negative emotion while controlling for the other three*.

* *p < 0.05*,

** *p < 0.01*,

*** *p < 0.001*.

**Figure 4:**
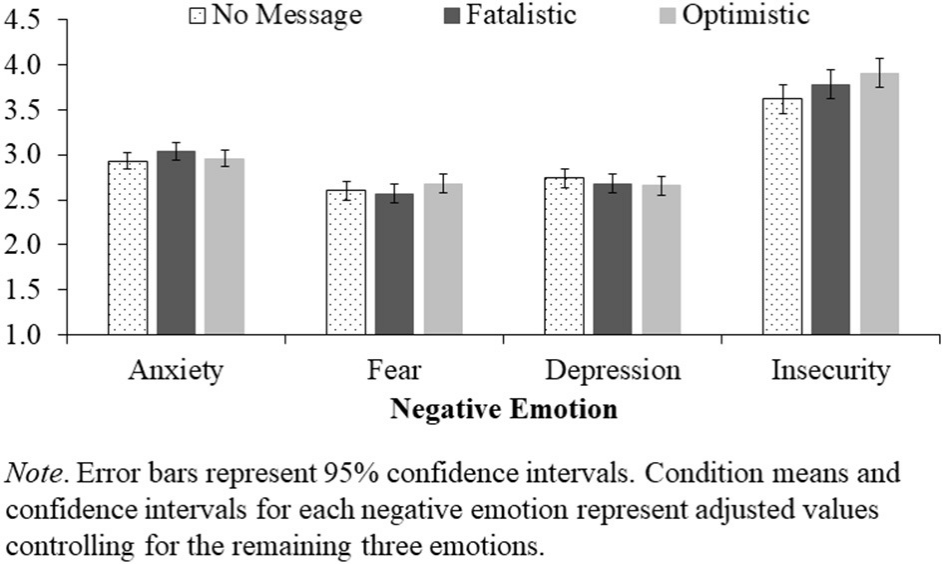
Effect of Messaging Condition on Emotional Distress.

**Figure 5:**
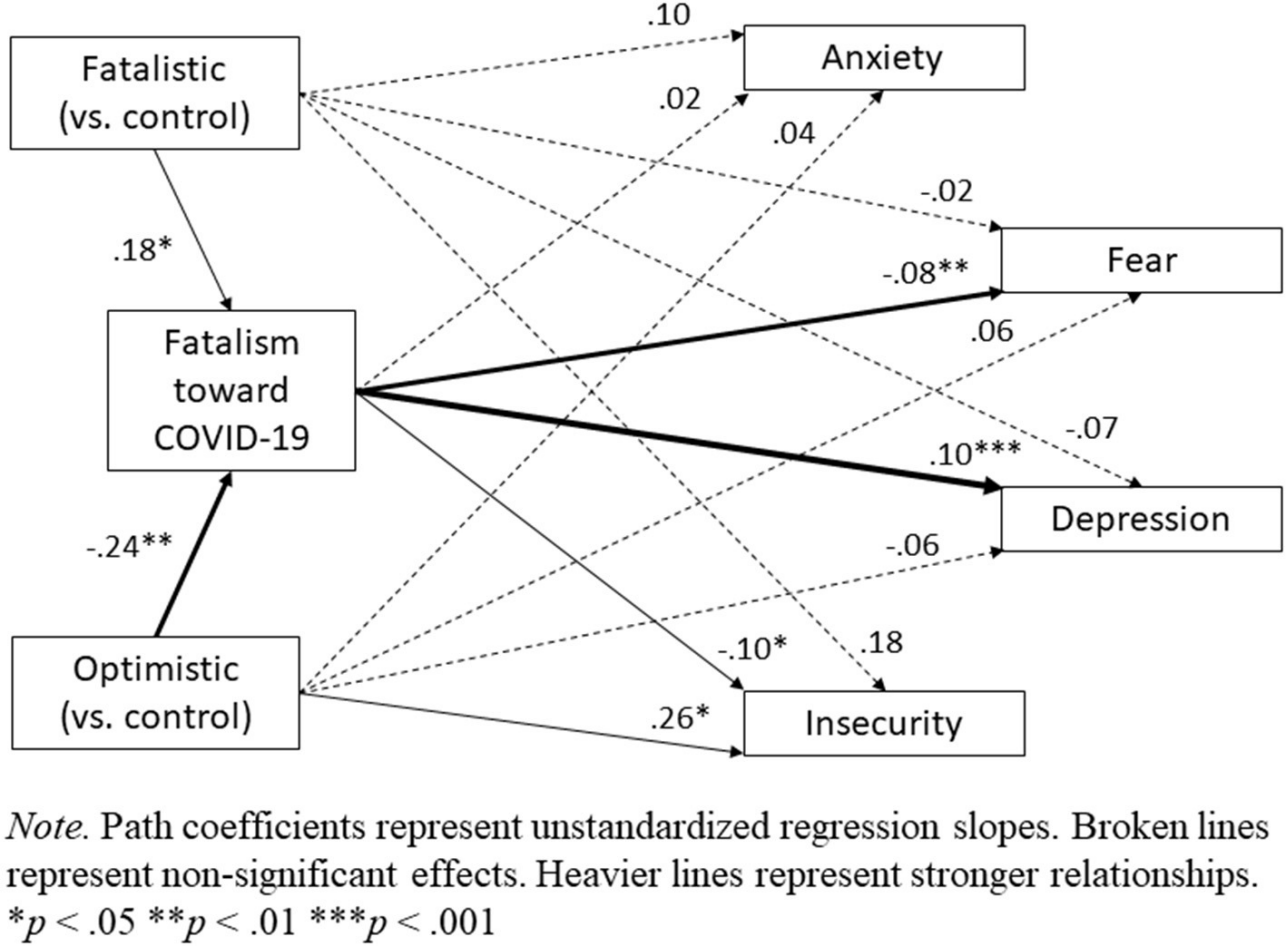
Path Model of Indirect and Direct Effects of Messaging Condition on Negative Emotions Through the Mediator of Fatalism toward COVID-19.

To examine the effect of messaging condition on negative emotionality, we first conducted four separate ANCOVAs on each emotion while controlling for the other three (removing the covariates did not affect these analyses, but see SOM for results without the covariates). These analyses revealed a significant effect of messaging condition on insecurity, *F*_(2, 845)_ = 3.21, *p* = 0.041, $\eta_{p}^{2}$ = 0.01, such that the optimistic message increased insecurity relative to no message, *t*(845) = 2.52, *p* = 0.012, *d* = 0.21, but the fatalistic message did not, *t*(845) = 1.43, *p* = 0.155, *d* = 0.12. There were no overall effects of condition for anxiety, *F*_(2, 845)_ = 1.33, *p* = 0.266, $\eta_{p}^{2}$ = 0.00, fear, *F*_(2, 845)_ = 1.14, *p* = 0.320, $\eta_{p}^{2}$ = 0.00, or depression„ *F*_(2, 845)_ = 0.61, *p* = 0.545, $\eta_{p}^{2}$ = 0.00 (see Figure 4). Nevertheless, given the significant effect of condition on fatalism and the significant partial correlations between fatalism and three of the four negative emotions, we tested for indirect effects of messaging condition on each emotion through the mediator of fatalism (as we did for support for mitigation) using Hayes' (2018) PROCESS macro (Model 4, 5,000 bootstrap resamples). A full path model is displayed in Figure 5, and indirect effects are summarized in Table 4 (see SOM for this analysis without the covariates). Overall, this analysis showed that by increasing fatalism toward COVID-19, the fatalistic message indirectly reduced fear and insecurity, but increased depression. The opposite pattern emerged for the optimistic message. By reducing fatalism toward COVID-19, the optimistic message indirectly increased fear and insecurity, but reduced depression.

**Table 4:** Indirect effects of messaging condition on negative emotions through COVID-19 Fatalism.

**Message Condition**	**Outcome**	**Effect**	**Boot SE**	**Boot LLCI**	**Boot ULCI**
Fatalistic	Anxiety	0.004	0.006	−0.004	0.022
(vs. control)	Fear	−0.015	0.009	−0.040	−0.002
	Depression	0.018	0.010	0.002	0.044
	Insecurity	−0.018	0.013	−0.056	−0.001
Optimistic	Anxiety	−0.006	0.007	−0.024	0.006
(vs. control)	Fear	0.019	0.010	0.004	0.045
	Depression	−0.023	0.012	0.004	−0.006
	Insecurity	0.024	0.015	0.003	0.063

*All indirect effects represent unstandardized regression coefficients. Boot, Bootstrapped; SE, Standard Error; LLCI, Lower Level Confidence Interval; ULCI, Upper Level Confidence Interval. Confidence intervals represent 95% CIs, thus intervals that do not contains zero are significant at the p < 0.05 level*.

## Discussion

The results were generally consistent with our hypotheses. They offer insights into the role of fatalism in the early days of the COVID-19 pandemic and show how media messaging may have influenced support for virus mitigation efforts and overall mental health.

First, as anticipated, the fatalistic message increased fatalism toward COVID-19 while the optimistic message reduced it. Results for behavioral intentions to support mitigation efforts were partially consistent with hypotheses. As predicted, the optimistic message increased support for mitigation, and fatalism toward COVID-19 mediated this effect. However, the fatalistic message showed no overall effect on support for mitigation. Nevertheless, consistent with hypotheses, this message reduced support for mitigation indirectly by increasing fatalism toward COVID-19. Interestingly, after accounting for this indirect effect, we found that the fatalistic message increased support for mitigation directly (see Figure 3). This pattern explains why the fatalistic message had no overall effect on support for mitigation—suggesting that the message produced two opposing effects (a suppression effect). Whereas, some people became fatalistic and thus less supportive of mitigation efforts, others reacted against the message by increasing their support for mitigation. This pattern may be indicative of reactance (Brehm, 1966), wherein people respond to external pressure by asserting their freedom and control. Although this interpretation would suggest that some identifiable moderator could predict who responded to fatalistic media messages with reactance (vs. fatalism), we found no significant moderators of this relationship in our data (see SOM for exploratory analyses). With that said, we included only a small number of personality scales in our study (self-esteem, BAS/BIS sensitivity). Future research could examine alternative personality factors (e.g., agreeableness), or other individual differences (e.g., personal experience or prior knowledge of viral epidemiology) that might moderate responses to fatalistic messages.

Fatalism toward COVID-19 also showed associations with emotional distress, and messaging condition evinced significant indirect effects on negative emotionality by influencing fatalism. First and foremost, fatalism was positively associated with depression. However, when controlling for the other negative emotions assessed in the study, fatalism was also negatively associated with fear and insecurity. These associations are only partially consistent with expectations. We hypothesized a positive association for depression given the withdrawal-oriented nature of fatalistically abandoning efforts to engage personal control (which was supported), but a negative association with anxiety given that fatalism can offer defense against intractable threats (Hayes et al., 2016). Results showed no associations between fatalism and anxiety (with or without the covariates). But the associations of fatalism with fear and insecurity may be consistent with our hypothesis that fatalism is an attempt to cope with the intractable nature of the pandemic. In retrospect, anxiety should be most likely when examining distant or abstract threats where the possibility of negative outcomes is highly uncertain (McNaughton and Corr, 2004). While this may have applied to the early days of the pandemic, it is only conceivable that most people saw COVID-19 as a clear and present danger. If so, it would be reasonable to expect fear-related emotions (Greenberg et al., 1997; McNaughton and Corr, 2004). These results would need to be replicated to ensure that they are reliable, but the associations with fear and insecurity are at least partially consistent with our expectation that fatalism toward COVID-19 offers a means of reducing concerns about the pandemic.

One aspect of the results that was not anticipated was the effect of the optimistic message on feelings of insecurity (see Figure 4). Results from the mediational analysis suggest that part of this effect is attributable to reduced feelings of fatalism (which is consistent with our theorizing about the relationship between fatalism and pandemic concerns), but the direct effect shows that the optimistic message increased feelings of insecurity even after controlling for the influence of fatalism (see Figure 5). One possible interpretation for this effect is that people who read the optimistic message became more vigilant about their health-status. They may have become less confident that they were safe and healthy, for instance, and more guarded against contracting the virus and potentially transmitting it to others. From this perspective, feelings of insecurity may be somewhat adaptive during a pandemic. While feeling insecure is no doubt emotionally taxing and likely difficult to maintain for extended periods, a certain level of distress may be necessary to remain vigilant against infection. Future research could investigate this possibility more directly.

### Implications for COVID-19 Mitigation

The results of the current study have important implications for ongoing efforts to mitigate the spread of COVID-19 by suggesting a pivotal role for fatalism. Indeed, several studies now show that fatalistic thinking reduces behavioral intentions to follow public health advice aimed at mitigating the spread of COVID-19. More specifically, high belief in predetermination (Özdil et al., 2021), exaggerated estimates of the infectiousness of the virus (Akesson et al., 2020), and the tendency to automatically associating the virus with death (Jimenez et al., 2020) have all be found to be associated with an unwillingness to follow mitigation protocols (see also Bogolyubova et al., 2021). In essence, people are unlikely to engage mitigation efforts unless they believe COVID-19 can be eradicated by such efforts and that their actions (e.g., social distancing, staying at home) are needed to stop the virus.

The current study suggests that media messaging plays an important role in affecting mitigation efforts by virtue of influencing fatalism toward COVID-19. Messages that paint a bleak picture of the pandemic, or suggest that it may take years to end (if it will end at all) may undermine support for mitigation efforts by promoting fatalism (cf., Briscese et al., 2020). Ironically, such messages may ultimately serve to prolong the pandemic and increase its severity by discouraging adherence to public health guidelines. By the same token, our results also suggest that messaging about the virus can be an important means of reducing fatalism and thereby increasing support for mitigation. Pro-mitigation messages that are inherently anti-fatalistic by drawing clear connections between individual actions and the spread of the virus have been shown to increase intentions to practice social distancing (Lunn et al., 2020). Moreover, messages that promote a duty to care for others (Everett et al., 2020) have also been found to be effective in promoting adherence to mitigation protocols, and these too may function in part by reducing fatalism (see SOM for associations between concern for others, fatalism, and support for mitigation).

### Implications for Mental Health

The current research also has implications for understanding the mental health consequences of the pandemic. Numerous studies have found increased prevalence of psychological disfunction stemming from the pandemic (e.g., Bo et al., 2020; Choi et al., 2020; Forte et al., 2020; Gallagher et al., 2020; Hyland et al., 2020; Salari et al., 2020; Shevlin et al., 2020). These studies point toward elevated levels of anxiety, depression, and trauma. The pandemic not only poses a threat to our physical health, but also increases the burden of everyday life while sapping the financial and psychological resources needed to cope with this burden. Indeed, people with direct personal experience with the disease (e.g., Forte et al., 2020; Gallagher et al., 2020), low or reduced income (e.g., Hyland et al., 2020; Shevlin et al., 2020), and those who are alone or detached from loved ones (e.g., Horesh et al., 2020; Parlapani et al., 2020) are among those reporting higher levels of psychological distress during the pandemic.

Studies suggests that at least some of the mental distress triggered by the pandemic stems from feelings of fatalism toward COVID-19. Specifically, Ngien and Jiang (2021) found that COVID-19 fatalism was positively associated with stress among Chinese youth, and Bogolyubova et al. (2021) found that fatalism predicted post-traumatic stress symptoms in an international sample. In the current study, we found that fatalism toward COVID-19 was positively associated with depression. Moreover, we found that media messaging can influence depression by affecting fatalism. In fact, our fatalistic message was partly inspired by extent media messages, including Donald Trump's tweet about the cure being worse than the disease. At the time of data collection (March 27th), the possibility that President Trump would forgo restrictions and allow the virus to go unmitigated to save the economy appeared real. We reasoned that this anti-mitigation rhetoric may be effective in reducing support for mitigation, but suspected it would also promote depression and despair by causing many people to feel fatalistic about the pandemic. Our results support this reasoning. And moreover, the data also show that optimistic media messaging can reduce feelings of depression by reducing fatalism toward the COVID-19 pandemic. Ngien and Jiang (2021) observed similar results showing that social media use reduced pandemic stress by virtue of reducing feelings of fatalism. Thus, the mental well-being of people who consume media related to the pandemic may hinge in part upon the extent to which the message makes them feel fatalistic (vs. powerful and effective) toward the virus.

Our data also add complexity to the mental health picture by showing that fatalism toward the pandemic can offer protection against fear and insecurity. These results are consistent with research by Özdil et al. (2021) who found that fatalistic beliefs relating to predetermination and luck were negatively associated with fear of COVID-19. Similarly, Lifshin et al. (2020) found that extremely high levels of helplessness toward becoming infected were associated with lower levels of anxiety. Thus, believing that nothing can be done to stop the virus or to prevent oneself from being infected precludes the need to worry about it. Indeed, we maintain that this is the inherent appeal of fatalism in response to intractable threats such as the COVID-19 pandemic. However, fatalism should not be viewed as a healthy solution to the problem. Indeed, the evidence suggest that fatalism is associated with reduced intentions to follow public health advice—behavior that poses a risk to oneself and others. Moreover, even the evidence suggesting that fatalism is associated with lower fear and anxiety shows that this is not without caveats. For instance, Özdil et al. (2021) found that at least one facet of fatalistic (pessimism) was associated with *more* fear of COVID-19. This may help to explain why we only found a negative association between fatalism and fear after controlling for other negative emotions (such as depression, which is strongly associated with pessimism). Likewise, Lifshin et al. (2020) found that moderate (vs. low) levels of helplessness were associated with *increased* anxiety (i.e., the relationship between helplessness and anxiety was curvilinear). According to Lifshin et al. (2020), moderate levels of helplessness may be associated with feeling overwhelmed by difficult circumstances that exceed one's capacity for control whereas extremely high levels of helplessness can offer relief from anxiety because there is truly nothing that can be done.

In our view, fatalism toward a specific phenomenon that is truly impossible to control can be an adaptive response under the circumstances. The trouble occurs when people turn to fatalism too quickly or the fatalistic giving-up process is too extensive (see Hayes et al., 2017). Becoming fatalistic too quickly can lead people to miss the chance to control something that ultimately *can* be controlled. In the context of COVID-19, fatalism in the first few weeks of the pandemic may have led humanity to miss the opportunity to minimize the global impact of COVID-19. Moreover, given what may be lost by giving-in to a pandemic (the health and survival of oneself and those to which one is connected), fatalism in the context of COVID-19 may be quite extensive and could trigger generalized fatalism that leads to severe depression and other mental health issues. Whatever the case may be, it appears that the emotional correlates of fatalism toward COVID-19 are complex and multifaceted. Future research should continue to investigate the role of fatalism in mental health outcomes to the pandemic and beyond.

### Important Limitations

Although this research is largely supportive of our hypotheses, it also has several important limitations. First, the size of the effects of our manipulation on COVID-19 fatalism, support for mitigation efforts, and emotional well-being are quite small. Indeed, according to rules of thumb for gauging the size of a standardized effect (Cohen's *d*), nearly all effect sizes observed in this study were small (<0.49) or very small (<0.20). These effects were detected as significant due to a relatively large sample size. Nevertheless, what may begin as a small effect at the beginning of a pandemic may snowball into much larger effects as time passes.

Second, the measures that we employed to test our hypotheses were not standardized instruments. It is therefore difficult to compare the scores observed in the current study with comparable scores in the existing literature. Did participants in our sample report particularly high levels of fatalism? Likewise, were the depression scores observed in our sample indicative of clinical depression or normal sadness? The reason that we did not use standardized measures of fatalism or support for mitigation efforts was that none existed at the time of data collection. Even now it is hard to know if the observed values are relatively high or low. Nevertheless, we can gain some perspective on the level of negative affect reported in the current study by comparing the current data to pre-pandemic studies in our lab that used similar methodology. For instance, Hayes and Hubley (2017) assessed anxiety and depression with the same items used in the current research (in addition to several others). Mean scores for anxiety were significantly higher in the current sample (*M* = 2.98; *SD* = 1.44; *n* = 851) than they were on the same items prior to the pandemic (*M* = 2.03; *SD* = 1.40; *n* = 204), *t*(1,053) = 8.45, *p* < 0.001, *d* = 0.66. Likewise, scores on the depression items were also significantly higher in the current sample (*M* = 2.69; *SD* = 1.69; *n* = 851), than before the pandemic (*M* = 2.07; *SD* = 1.67; *n* = 204), *t*(1,053) = 4.82, *p* < 0.001, *d* = 0.38. Thus, the levels of anxiety and depression reported in this study are significantly above what we have observed in our previous research. This is consistent with several studies that *did* use standardized measures and also found increased anxiety and depression in response to the pandemic (e.g., Hyland et al., 2020; Özdin and Özdin, 2020; Shevlin et al., 2020). To provide further contextualization to the depression scores in our sample, Hayes and Hubley (2017) also assessed depression using the CESD-10 (Andresen et al., 1994) together with the same 5-items used to assess depression in the current study. Scores on the 5-item state depression scale were highly correlated with scores on the CESD-10 in this previous study, *r*(204) = 0.76, *p* < 0.001. As such, while we cannot offer firm conclusions about the severity of negative affect observed in this study, the available evidence suggests that participants were experiencing abnormally high levels of anxiety and depression.

## Conclusion

The COVID-19 pandemic has been a shock to nearly everyone on the planet. The early stages may have been among the most stressful and uncertain. The threat of potential infection and death coupled with seemingly irreparable disruptions to nearly every aspect of everyday life represents a burden of momentous proportions. The current research suggests that fatalistically giving up may have helped to quell some of the fear and insecurity aroused by the pandemic. Feeling that one cannot possibly change the situation may offer some relief from a situation that demands constant vigilance and control. But fatalism is also strongly associated with depression, so fatalistically withdrawing from efforts to cope with the pandemic is not without emotional costs (cf., Hayes et al., 2016).

Critically, fatalism toward COVID-19 was also found to promote giving up on public health regulations that function to protect oneself and others. Fatalism in the face of COVID-19 is thus self-destructive and a public health liability. Unfortunately, media messages—some of which came directly from prominent authority figures—only served to promote fatalism in the early stages of the pandemic. The cost of early failures to mitigate the spread of a deadly virus cannot be overstated.

When faced with future pandemics, the current research suggests that early interventions aimed at preventing (rather than promoting) fatalistic thinking might be among the most important means of promoting adherence to mitigation protocols and reducing depression. Recognizing that people may be drawn toward fatalism to reduce fear and insecurity might be equally important. Offering alternative means of coping with these negative affective states—with public policy and/or consistent optimistic media messaging—may be an effective means of preventing fatalism from taking hold.

## Data Availability Statement

The dataset presented in this study can be found in an online repository: Open Science Framework, https://osf.io/sx7g2/.

## Ethics Statement

This study was reviewed and approved by the Acadia University Research Ethics Board. Participants provided informed consent prior to participating in the study.

## Author's Note

The materials used in the study, as well as supplemental data analyses are publicly available at https://osf.io/sx7g2/. De-identified data and analysis script are also posted at the same link.

## Author Contributions

JH and LC developed the study concept. JH designed the study, and LC provided critical feedback. Data collection, analysis, and interpretation was performed by JH. LC performed an extensive review of the literature. JH drafted the manuscript, and LC provided critical revisions. All authors approved the final version of the manuscript for submission.

## Conflict of Interest

The authors declare that the research was conducted in the absence of any commercial or financial relationships that could be construed as a potential conflict of interest.
